# Lingual necrosis secondary to group a streptococcal necrotising infection

**DOI:** 10.1093/omcr/omaf022

**Published:** 2025-04-28

**Authors:** Fatima Abeer, Madan Ethunandan, Sara Waise, Kordo Saeed

**Affiliations:** Department of Intensive Care Medicine, University Hospital Southampton, Tremona Road, Southampton, Hampshire SO16 6YD, United Kingdom; Department of Oral and Maxillofacial Surgery, University Hospital Southampton, Tremona Road, Southampton, Hampshire SO16 6YD, United Kingdom; Department of Histopathology, University Hospital Southampton, Tremona Road, Southampton, Hampshire SO16 6YD, United Kingdom; Department of Microbiology, University Hospital Southampton, Tremona Road, Southampton, Hampshire SO16 6YD, United Kingdom

**Keywords:** case report, group a streptococcal infection, lingual necrosis, necrotising soft tissue infection

## Abstract

Tongue necrosis is a rare manifestation of Group A Streptococcal (GAS) infection. Only certain strains of GAS have been found to cause invasive disease in the form of necrotising skin and soft tissue infections. We present the case of an older woman who developed a sore throat and localised ulcer on the tongue, that rapidly progressed to lingual necrosis and spreading erythema in the neck and chest. Owing to rapid detection, she received timely surgical treatment and intravenous antibiotic therapy, making a full recovery. This report describes the unique presentation of lingual necrosis in GAS infection. It highlights the diagnostic challenges and emphasises the importance of early recognition of symptoms.

## Introduction

Giant cell arteritis, ischaemia, and vasculitis are the most common diseases associated with tongue necrosis [1]. The tongue and other facial structures are rarely reported to be affected by Group A Streptococcal (GAS) infection. The primary lineage associated with invasive disease in Europe and the USA is the GAS emm1 strain [2]. Research suggests that mutations within this strain, can increase its virulence and transform it into a superantigen. These mutations have been found in cases of giant cell necrosis, haemorrhagic pneumonia, and streptococcal toxic shock [2].

## Case report

A 60-year-old woman presented with a 3-day history of sore throat and a localised ulcer on the right side of the tongue. She applied benzocaine cream to the tongue for pain relief but subsequently developed progressive swelling of the tongue, as well as odynophagia and trismus. Her medical history included psoriatic arthritis controlled with etanercept and a dental root extraction performed 1 month previously.

**Figure 1:**
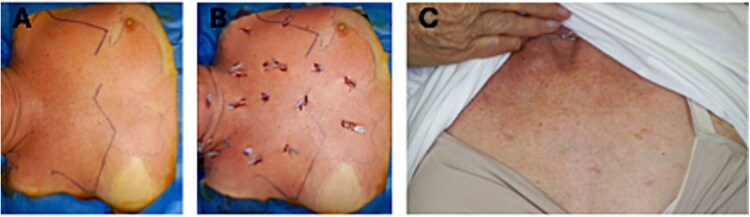
Stages of progression of chest wall erythema. (A) Extensive erythema spreading across the neck and chest. (B) Chest decompression with multifocal drainage points. (C) Appear 4 months after initial presentation.

On presentation to the emergency department, she was hypotensive and had a blood pressure of 80/50 mmHg and a heart rate of 100 beats/min. She was initially treated for anaphylaxis with two doses of adrenaline and 3 litres of fluid boluses. However, because she was unable to maintain her blood pressure, metaraminol was required for cardiovascular support. She was afebrile and maintained 95% oxygen saturation with 2 L of oxygen. Wheezing, stridor, or immediate airway compromise was not observed. Physical examination revealed right-sided lymphadenopathy and minimal erythema over the anterior chest wall (Fig. 1).

**Figure 2:**
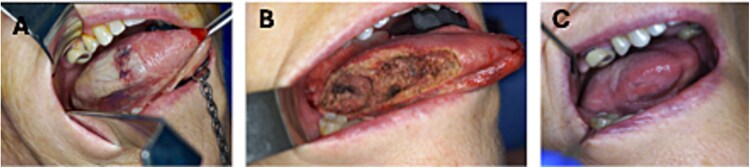
Stages of progression of lingual necrosis: (A) ulcerated, necrotic and ischaemic area of the tongue (necrotising fasciitis/myositis). (B) Right partial glossectomy defect after debridement. (C) Completely mucosalised tongue 4 months after initial presentation.

Fibreoptic nasoendoscopy revealed significant swelling in the right hypopharynx and submandibular area extending to the upper neck, with patent airway and trachea. There was significant bluish-brown discolouration and swelling of the tongue, accompanied by shedding of the superficial epithelium (Fig. 2). Elective endotracheal intubation was performed due to the high risk of airway compromise.

Head and neck computed tomography (CT) showed no radiological features of fulminant or gas-producing necrotising fasciitis. Neither mediastinitis nor deep neck space collection were evident. However, there was reduced attenuation and poor enhancement of the tongue mucosa. Laboratory tests were normal except for relative leukopenia with a white blood cell count of 2.6 × 10^9^/l and an elevated C-reactive protein level of 132 mg/l.

She was admitted to the intensive care unit with suspected lingual vasculitis and a secondary infection. However, admission blood cultures revealed a GAS infection, specifically with the emm1 strain. The maxillofacial team promptly performed an emergency debridement in the form of a partial right glossectomy, and incision and decompression of the neck and chest. Early diagnosis and timely intervention made it possible to avoid neck and chest skin excision.

**Figure 3:**
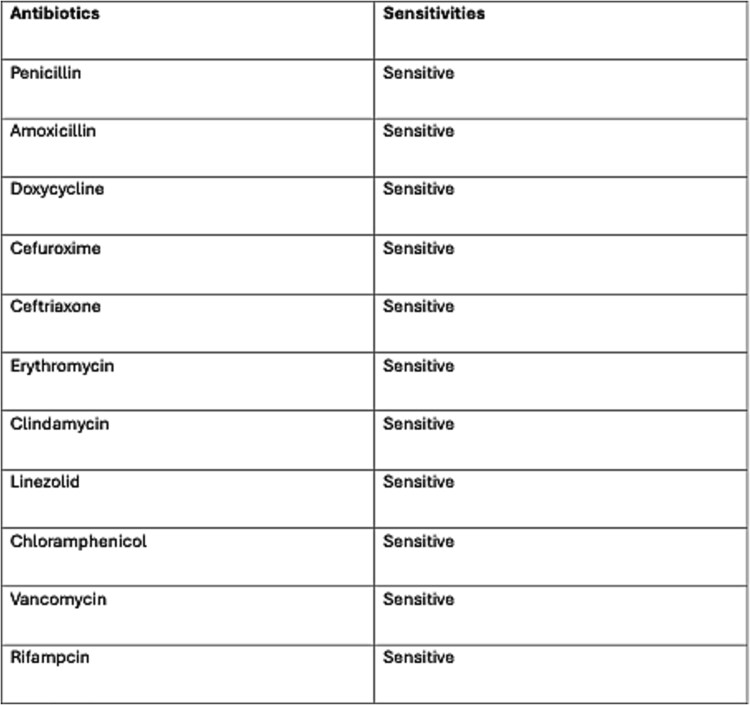
Antibiotic susceptibility report for Group A streptococcus (GAS) blood culture, showing susceptibility to all tested antibiotics.

Post-operatively she remained intubated for 9 days and was managed in the intensive care unit. There she underwent a 6-day course of co-amoxiclav, followed by a 5-day course of benzylpenicillin and clindamycin for targeted therapy (Fig. 3). She underwent two additional planned examinations and washouts under anaesthesia as part of her comprehensive management plan.

After 17 days of hospitalisation, she was discharged home. At the 4-month follow-up, she was in good condition, showing no evidence of residual swelling or erythema. The tongue defect had completely mucosalised, and there was no loss of skin on the neck or chest.

## Discussion

GAS is one of the most commonly isolated organisms in patients with necrotising fasciitis [3].

Risk factors that predispose people include diabetes mellitus, chronic renal disease, alcoholism, and an immunocompromised state [4]. It arises from trauma or distant haematogenous spread. In this case, immunosuppression due to etanercept treatment was a significant risk factor.

The diagnosis of necrotising soft tissue infection (NSTI) is challenging and depends on the judgement and experience of clinicians. Swelling, erythema, and disproportionate pain should raise suspicion of NSTI. The presence of blisters, bullae, and skin crepitus is a strong indication [5]. Most infections do not result in skin or soft tissue necrosis; however, those that do are associated with a high mortality rate [5]. In immunocompromised patients, physical examination findings are often subtle, and laboratory results are non-specific, further complicating the diagnostic process. Nevertheless, ecthyma gangrenosum and haemorrhagic pustules developing into necrotic ulcers are the most common signs of necrotising infections in these individuals [6].

Surgical exploration remains the gold standard for NSTI diagnosis. Early and comprehensive surgical debridement is crucial to control the infection [5]. Delays in initial debridement are often linked to poorer outcomes, whereas immediate surgery typically results in a shorter stay in intensive care and hospital [5]. In this case, timely surgical debridement limited the necrosis to the tongue, and spared the overlying skin.

CT scans can be helpful in diagnosing NSTI. However, less than half of CT patients show gas tracking in the fascial planes [7], a characteristic feature for the diagnosis of necrotising infection.

The first line antimicrobial agent for GAS infection is penicillin. The patient should initially be treated with broad-spectrum antibiotics, followed by targeted therapy if a specific pathogen is identified. Clindamycin is a valuable adjunct to penicillin and offers numerous benefits in the treatment of necrotising fasciitis and toxic shock syndrome [5]. The optimal duration of treatment is not clearly defined, but should continue until surgical debridement is complete, clinical improvement is achieved, and fever has resolved for 48–72 h [5].

This case highlights the need for greater awareness of NSTIs among healthcare professionals. For clinicians, immediate surgical referral and early antibiotic therapy are of paramount importance for potentially saving patients’ lives.
